# A Large Dentigerous Cyst in a Child as a Complication of Deciduous Molar Endodontic Treatment: An Interesting Case Report with Short Literature Review

**DOI:** 10.1155/2023/4406854

**Published:** 2023-05-08

**Authors:** Naida Hadziabdic, Amila Balic, Emina Cengic, Elma Katana, Damir Duratbegovic, Edina Lazovic Salcin

**Affiliations:** ^1^Department for Oral Surgery and Dental Implantology, Faculty of Dentistry with Dental Clinical Center, University of Sarajevo, Sarajevo, Bosnia and Herzegovina; ^2^Department for Preventive and Pediatric Dentistry, Faculty of Dentistry with Dental Clinical Center, University of Sarajevo, Sarajevo, Bosnia and Herzegovina; ^3^Department of Pathology, Faculty of Medicine, University of Sarajevo, Sarajevo, Bosnia and Herzegovina

## Abstract

This study presents a case report of an inflammatory dentigerous cyst of tooth #35, associated with its previously endodontically treated deciduous predecessor. Cystic lesion growth caused impaction of the second premolar, displacing it closer to the lower border of the mandible. The lesion represents a typical dentigerous cyst that possibly arises in association with periapical inflammation of a deciduous molar involving the follicle of the premolars. This report highlights the inflammatory etiology of dentigerous cysts, which mainly occur in mixed dentition. A 12-year-old patient was referred to Oral Surgery Department regarding a sizeable radiolucent lesion in the unerupted mandibular second premolar region, detected on an Orthopantomagram (OPG) X-ray. A non-vital primary predecessor had been endodontically treated at least one year before an examination, with a control OPG X-ray showing no signs of pathology at the time. The patient did not report any symptoms. Clinical examination revealed an egg-like tumefaction of the alveolar bone in the left premolar region of the mandible. Cone-beam computed tomography analysis showed a sizeable translucent lesion surrounding the crown of the impacted tooth. The lesion was enucleated in its entirety, along with the impacted premolar, under local anesthesia. Clinical findings combined with radiographic and microscopic examinations confirmed the diagnosis of an inflammatory dentigerous cyst. The seventeen month follow-up revealed good bone healing. This case presented a rare complication of endodontic treatment of deciduous teeth and informed on possible complications of endodontic therapy in deciduous teeth, emphasizing the importance of early diagnosis of cysts in preventing extraction of permanent teeth.

## 1. Introduction

Odontogenic cysts are described as a heterogeneous group of cystic lesions of the mouth, originating from the tissue of the dental organ. They are the second most common cystic lesion of the jaws, radicular cysts being the most prevalent ones. All odontogenic cysts combined comprise up to 24% of all true cysts of the jaws, making them the most common type of oral cyst to be found in children [1]. Being classified as true cysts of the jaws, these cystic lesions are characterized by having an outer fibrous cystic shell, lined with epithelium on the inner surface of the membrane. Dentigerous cysts can be easily distinguished by their attachment to the cemento-enamel junction of an unerupted or impacted tooth, with its crown protruding into the cystic cavity [2]. They originate from altered tissue of the reduced enamel epithelium of the follicle, always occurring after completed amelogenesis. These changes are followed by the secretion of the cystic fluid into the space between the follicle and the crown of the permanent tooth, causing a continuous growth of the cyst. They usually involve mandibular third molars, maxillary canines, maxillary third molars, mandibular premolars, as well as supernumerary teeth, such as mesiodens [1, 3]. Despite being considered benign lesions, the literature reports examples of neoplastic alterations in the tissue of dentigerous cysts, leading to ameloblastoma development [4].

The exact etiopathogenesis of these cysts still remains unclear [1, 5]. Benn and Altini were the first to propose the classification of dentigerous cysts based on their etiology, by dividing them into two separate groups: dentigerous cysts of developmental and inflammatory origin [1]. According to one of the theories explaining their origin, these cysts can arise due to the propagation of an inflammatory process from the periapical region of non-vital, destructed, or previously endodontically treated primary teeth, into the follicular tissue of its permanent successor, with these structures being in close proximity of one another [6]. As a result, the periapical inflammatory process acts as a trigger, causing follicular tissue alteration and the potential formation of the cyst. Literature shows a series of case reports stating an obvious relation between dentigerous cyst formation and endodontic treatment of a tooth in its close proximity, often being the primary predecessor of the affected tooth [1, 6].

They frequently arise in mixed dentition, during the first years of the second decade, more often in boys than in girls [6]. They are most commonly located in the premolar region, which, in many cases, still contains carious and non-vital primary molars. Smaller cysts are usually asymptomatic, with no signs or symptoms in patients, and are discovered during a radiologic examination of impacted teeth [3, 7]. The cyst growth is followed by intraoral swelling of the affected region, which frequently results in the dislocation of the impacted tooth. Radiologic findings reveal a unilocular radiolucency in the jaw, resulting in permanent tooth dislocation and impaction, with its non-vital successor still present in the dental arch [1].

This case report shows a clinical finding of a dentigerous cyst of a tooth, related to its previously endodontically treated primary predecessor. The aim of this report is to point out this possible negative outcome of endodontic treatment in primary teeth, potentially leading to the loss of permanent teeth. It is important to highlight the importance of periodic control examinations of endodontically treated primary teeth, with the goal to prevent such negative outcomes.

## 2. Case Report

### 2.1. Medical History and Clinical Examination

The patient, a twelve-year-old girl, was referred to the Clinic of Oral Surgery by her orthodontist, due to a suspected cystic lesion in the mandible routinely discovered on the Orthopantomagram (OPG) X-ray. The patient had previously denied having any subjective symptoms. Clinical examination revealed a significant swelling in the vestibule of the left premolar mandible region between teeth #34 and #36. These findings indicated the presence of a relatively big cystic lesion. There was no visible extraoral deformity, which is why neither the patient's parents nor the patient herself noticed anything unusual.

#### 2.1.1. Radiologic Findings

**Figure 1:**
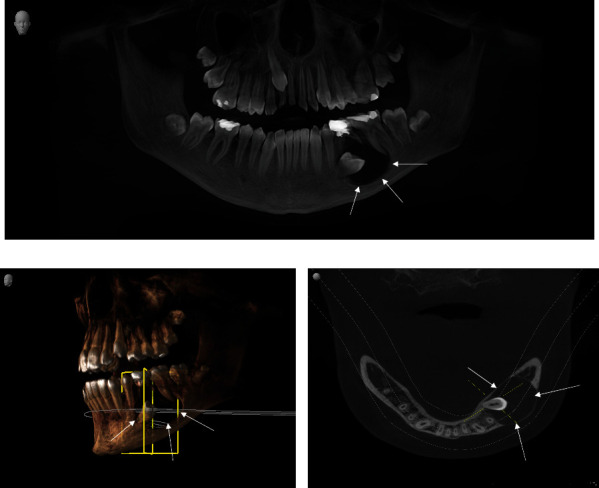
(a) OPG X-ray: unilocular radiolucency surrounding the crown of the impacted second premolar tooth #35, with its crown protruding into the cystis cavity. Border of the cystic lesion is in continuity with lamina dura of the tooth #75. (b) 3D CBCT: cystic lesion in relation to other anatomical structures of the mandible. (c) CBCT: coronal view of the cystic cavity.

Analysis of the OPG X-ray showed a round radiolucency in the left premolar region of the mandible (Figure 1(a)). The cystic lesion was primarily located in the body of the mandible, with a tendency of spreading to the lower margin of the jaw. Unresorbed roots of the previously endodontically treated deciduous second molars were in direct contact with the cystic cavity, including a second premolar's crown protruding into the cavity. Cone-beam computed tomography (CBCT) analysis showed the presence of a sizeable radiolucent lesion, its dimensions being 23.93 mm × 17.24 mm, with the cyst containing the crown of an impacted permanent tooth #35 (Figures 1(b) and 1(c)). The cystic cavity had communication with the mesial root of the first molar, which was also noted. Both cold and electric pulp tests confirmed the vitality of tooth #36. A working diagnosis of dentigerous mandibular cyst in the teeth #34–#36 region was made based on previously stated clinical and radiographic findings.

**Figure 2:**
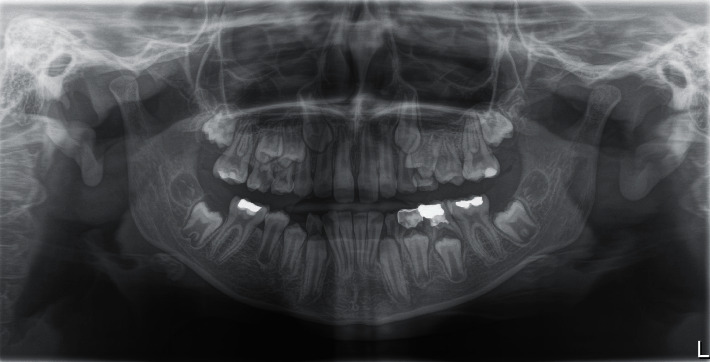
OPG X-ray taken during routine check-up of the patient, two years prior to the diagnosis of the cyst, showing no signs of pathology in left premolar region of the mandible at the time.

It is worth pointing out that an OPG X-ray was made two years prior to this diagnosis, and had shown no signs of pathology, with normal and expected position of both teeth, the primary second molar, and its secondary successor (Figure 2).

### 2.2. Treatment Protocol

**Figure 3:**
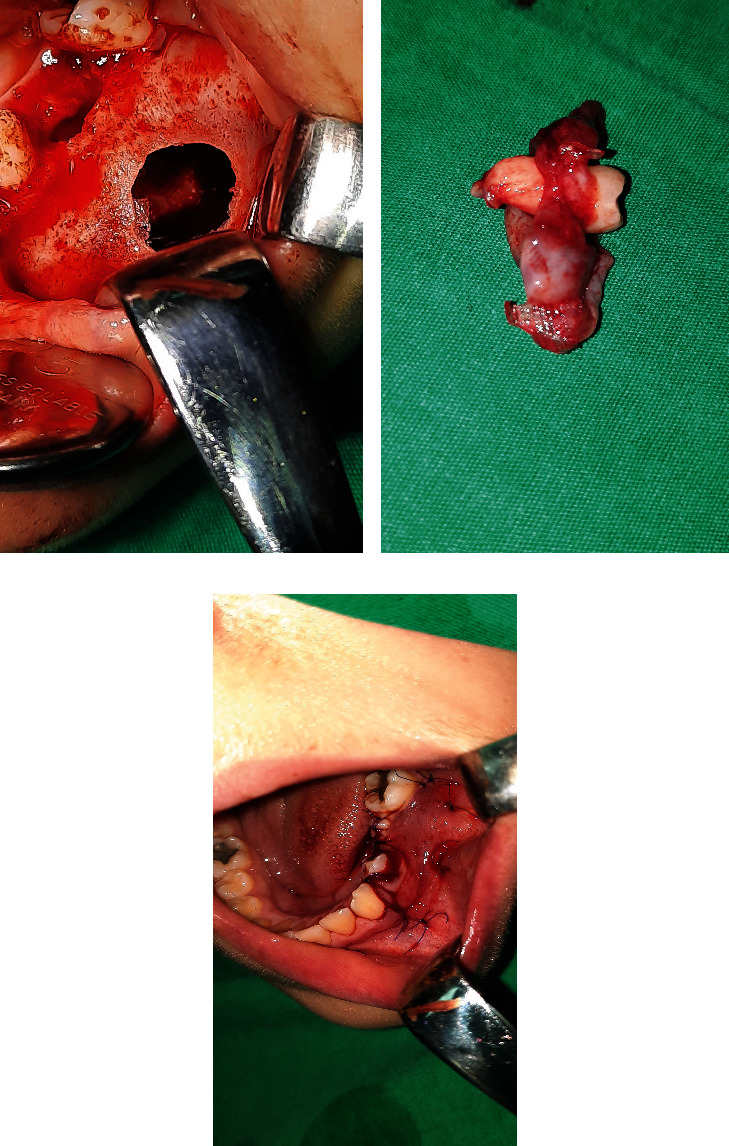
(a) Bony defect in the body of the mandible, after cyst enucleation. (b) Enucleated cyst, containing extracted permanent tooth. (c) Repositioned flap after placing two apical mattress sutures.

Following the administration of the local anesthetic, two vertical incisions were made in the region of teeth #33 and #36. A trapezoid mucoperiosteal flap was carefully raised. Upon direct inspection, the vestibular bone was found to have a round, ball-like deformity. After the deciduous molar was extracted, the bur was utilized to remove the thinnest part of the vestibular bone. A clear, yellow cystic fluid was aspirated. The surgical protocol contained total enucleation of the cyst, including the impacted permanent second premolar. After the wound irrigation was done, the mucoperiosteal flap was advanced, and two apical mattress sutures were placed. A complete cystic lining containing a permanent tooth was then sent for histopathological examination (Figure 3).

### 2.3. Patient Instructions

Postoperative medications included analgesics and antibiotics. The patient did not report any significant pain or complications during recovery. Due to a sizeable bone defect caused by the expansive growth of the cyst, orthodontic therapy was postponed until proper bone healing. In summary, the plan was to place a space maintainer to save room for a future implant.

### 2.4. Histopathological Findings

**Figure 4:**
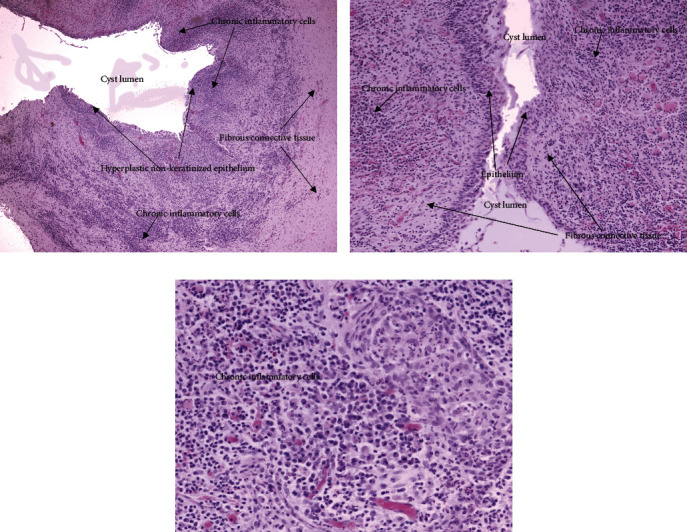
Microscopic findings of the sample Hematoxylin and Eosin (H&E). (a) Low-powered magnification (10×). (b) Cystic cavity lined by hyperplastic non-keratinized epithelium. (c) Chronic inflammatory cells in fibrous connective tissue of the cystic capsule.

Microscopic analysis of the sample material confirmed the diagnosis of a dentigerous cyst. It was also noted that the fibrous cystic wall contained an infiltrate with a large number of chronic inflammatory cells. The epithelium of the luminal surface of the fibrous cystic shell was classified as hyperplastic squamous epithelium without keratinization (Figure 4).

### 2.5. Control Examination

**Figure 5:**
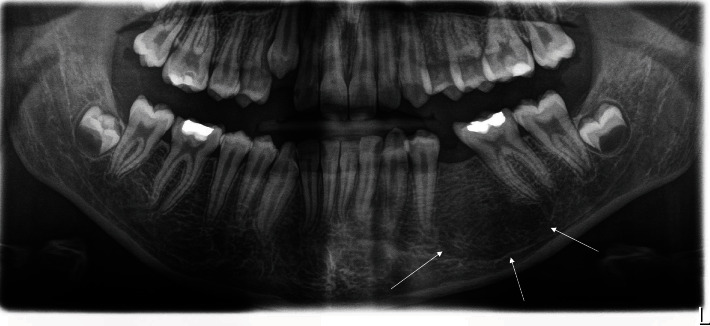
Control OPG X-ray showing good bone healing, with no signs of relapse.

A control examination was made one year after the surgery. The patient did not report any symptoms. Clinical and radiologic examination showed good bone healing, with no signs of relapse (Figure 5).

## 3. Discussion

Dentigerous cysts originate from the tissue of tooth follicles and are the most commonly diagnosed ones among all cysts of developmental origin [3]. They arise in both jaws, although mainly in the mandible [5, 8].

There are a few theories explaining the origin of dentigerous cysts. Even though their precise etiopathogenesis still remains unclear, they are mainly thought to be of developmental origin [1, 2]. It is suspected that the accumulation of cystic fluid in the space between the reduced enamel epithelium and the crown of the tooth leads to cyst formation. According to Main [9], accumulation of the fluid occurs due to the physical pressure exerted on the dental follicle by the tooth in eruption, causing obstruction in venous blood flow, leading to transudation of serum through the walls of capillary vessels. As a consequence of serum transudation, a change in capillary permeability occurs, leading to the rise of protein concentration in the cystic fluid [2]. According to Toller, a rise in osmolarity caused by the death of follicle epithelial cells leads to fluid accumulation and the formation of the cyst [10].

Bloch-Jorgensen was the first to propose inflammation as a potential trigger of dentigerous cyst formation. A series of case reports published by Bloch-Jorgensen showed the relationship between dentigerous cysts in primary teeth with their carious and necrotic primary predecessors [11]. According to this theory of origin, the inflammatory process surrounding the apex of the necrotic deciduous tooth spreads into the region of adjacent follicular tissue, causing epithelial alteration of the tissue. Due to changes in the epithelium, a secretion and accumulation of fluid occur, leading to cyst growth and expansion.

Azaz and Sheyter published a mini-series of five cases of dentigerous cysts forming around the mandibular second premolar, with apices of necrotic deciduous teeth in their close proximity [12]. They suspected that the inflammatory process surrounding the roots of deciduous teeth causes a continued, chronic irritation of the follicular epithelium of permanent teeth. Shaw et al. reported a series of 13 cases of cyst occurrence around the premolars, whose deciduous predecessors were also severely damaged by caries [13].

Based on these clinical reports, authors Benn and Altini proposed categorizing dentigerous cysts into two groups based on their origin: developmental and inflammatory dentigerous cysts [1]. According to these authors, developmental dentigerous cysts arise only during the period of permanent dentition, in the second and third decades of life. They are mostly found surrounding unerupted third molars. Inflammation of these cysts happens only as a result of cyst infection. On the other hand, dentigerous cysts of inflammatory origin arise from the tissue of the dental follicle, as a result of an inflammation spreading from the apical region of the adjacent primary tooth, most often being their necrotic primary predecessor. They commonly form around the mandibular premolar crown during mixed dentition. Microscopic examination revealed non-keratinized hyperplastic squamous epithelium with an abundance of chronic inflammatory cells in the cyst's fibrous capsule.

Dentigerous cysts of inflammatory origin usually occur in the mandible, with the mandibular second premolars being the most frequently affected teeth of the jaw [14, 15]. According to Lustig et al., this incidence happens due to two main reasons: mandibular deciduous molars are more susceptible to caries and necrosis than other teeth, and the fact that the roots of deciduous molars in the lower jaws happen to be in close proximity with the dental follicles of their permanent successors. Therefore, it is easier to suspect the spreading of the inflammatory process from the periapical regions of primary molars to adjacent anatomic structures, then often being follicles of permanent premolars [16].

This case report discusses the diagnosis of a dentigerous cyst in a child patient during the mixed dentition period. The affected tooth in this case, the mandibular second premolar, as well as the distinct microscopic finding of an abundant chronic inflammatory cell infiltrate in the fibrous capsule of the cyst, supports the theory of the inflammatory origin of these cysts, first proposed by authors Benn and Altini [1]. Radiographic examination showed the presence of a previously endodontically treated mandibular second deciduous molar, with its un-resorbed roots surrounded by a sizeable unilocular radiolucency, occupying the space normally taken by the follicle of the second permanent premolar. The sclerotic border of a radiolucent lesion appeared attached to the cemento-enamel junction of a dislocated and impacted second premolar, with its crown protruding into the cavity. It is worth noting that, two years prior to the diagnosis, a routine OPG X-ray had been taken, showing no signs of pathology at the time. This OPG X-ray also showed an endodontically treated deciduous molar, with its successor still being in the normal position in the bone. Grundy et al. reported that approximately 20 months pass before the diagnosis of inflammatory dentigerous cyst is made [17]. Histopathologically, a radicular cyst can resemble a dentigerous cyst, especially when there are few inflammatory cells present. A definitive diagnosis requires the correlation of clinical and radiological findings. The distinction is that a dentigerous cyst develops around an unerupted or impacted tooth (as seen in Figure 1), which we used to make the diagnosis, whereas a radicular cyst develops as a result of inflammation of a non-vital tooth. It should be noted that a radicular cyst can form around the roots of a non-vital deciduous tooth, but this is extremely rare [18, 19]. According to Benn and Altini's third histogenetic mechanism, we believe that, in our case, periapical inflammation from a non-vital deciduous tooth spreads to a permanent successor follicle, causing dentigerous cysts to form as a result of inflammatory exudate [1].

The infection, as a possible etiological factor of the cyst, in our case could originate as a consequence of the residual necrotic pulp tissue from the root canal of a previously pulpotomy-treated deciduous second molar. Pulpotomy was not performed at our clinic, and we were unable to obtain information on where and with what material (method) the pulpotomy was conducted.

Devitalization pulpotomy in two stages is still the most commonly used technique in our region for the treatment of pulp chronic infections in primary teeth. In the first session, devitalization paste based on paraformaldehyde is applied on the coronal pulp exposure, and in the second session after coronal pulp removal, antiseptic (zinc oxide eugenol paste and iodoform paste) or mummifying paste (based on parachlorophenol and camphor) is applied to the canal entrances. We cannot rule out the possibility that some of the materials used for the pulpotomy were the trigger for the cyst formation [5]. Other vital pulpotomy methods (formocresol, ferric sulphate, and Mineral trioxide aggregate (MTA)) are rarely used in our region.

Faugeron et al. and Petel et al. found no evidence to identify one superior pulpotomy medicament and technique clearly among ferric sulphate, formocresol, and MTA method [20, 21]. The residual necrotic pulp in root canals of pulpotomised teeth is a potential source of inflammation and side effects of pulpotomy, regardless of which pulpotomy method or material is used. According to the literature, the two foremost reasons for pulpotomy failures in primary teeth are improper diagnosis of radicular pulp inflammation status prior to treatment and pulp contamination due to micro-leakage [22].

Pulpectomy and lesion sterilization/tissue repair are appropriate methods for non-vital pulp treatment for primary teeth diagnosed with irreversible pulpitis or necrotic pulp [23].

Keeping the preceding in mind, it is also important to highlight the necessity of maintaining appropriate medical records and having them readily available because we cannot be certain that the cyst emergence was not caused by the irritating effect of some of the materials used. In order to detect possible complications on time, we emphasize the importance of continuous clinical and radiographic monitoring of all endodontically treated primary teeth.

There are a couple of different treatment approaches for dentigerous cysts proposed in the literature [24]. Conservative approach implies extraction of the affected primary tooth, leaving the possibility of spontaneous eruption of its permanent successor. This approach considers decompression and marsupialization of the cyst, with cystic tissue remaining in the jaw [1]. A more radical approach is used in cases of expansive growth of the cyst, which often causes severe dislocation and impaction of the permanent tooth, with no possibility of its spontaneous eruption. This treatment option consists of extraction of the affected primary tooth and total enucleation of the cyst, including the impacted permanent tooth [16].

Based on clinical and radiological findings, the prognosis of a spontaneous eruption of the impacted tooth in this case was very poor, due to its severe dislocation and angulation in the bone. Therefore, the dentigerous cyst was totally enucleated, including the impacted second premolar. Control checkup after seventeen months showed no signs of recurrence, with good bone healing.

The aim of this report was to highlight a potential complication of endodontic treatment of deciduous teeth, which can lead to the loss of permanent teeth. Improper endodontic treatment of the primary tooth indicates tooth extraction, with recommended periodic follow-up until an eruption of its permanent successor is finished. Therefore, it is of utmost importance to timely spot any changes in the expected eruption of primary teeth. The prolonged presence of primary teeth with no signs of root resorption should be evaluated by radiologic examination. Parents should be made aware of this potential complication of primary tooth endodontic treatment in order to recognize obvious signs and symptoms indicating the formation of cystic lesions in children.

## Data Availability

Data supporting this research article are available from the corresponding author or first author on reasonable request.
